# Review of Different Sequence Motif Finding Algorithms

**Published:** 2019

**Authors:** Fatma A. Hashim, Mai S. Mabrouk, Walid Al-Atabany

**Affiliations:** 1. Department of Biomedical Engineering, Helwan University, Egypt; 2. Department of Biomedical Engineering, Misr University for Science and Technology (MUST), Egypt

**Keywords:** Algorithms, Bioinformatics, Consensus, Gene expression regulation, Nucleotide motif, Protein binding

## Abstract

The DNA motif discovery is a primary step in many systems for studying gene function. Motif discovery plays a vital role in identification of Transcription Factor Binding Sites (TFBSs) that help in learning the mechanisms for regulation of gene expression. Over the past decades, different algorithms were used to design fast and accurate motif discovery tools. These algorithms are generally classified into consensus or probabilistic approaches that many of them are time-consuming and easily trapped in a local optimum. Nature-inspired algorithms and many of combinatorial algorithms are recently proposed to overcome these problems. This paper presents a general classification of motif discovery algorithms with new sub-categories that facilitate building a successful motif discovery algorithm. It also presents a summary of comparison between them.

## Introduction

Motif discovery is one of the sequence analysis problems under the application layer and it is one of the significant difficulties in bioinformatics applications. A DNA sequence motif is a subsequence of DNA sequence that is a short similar recurring pattern of nucleotides, and it has many biological functions 1. A DNA motif refers to a short similar repeated pattern of nucleotides that has biological meaning. Sequence motifs also called regulatory elements exist in Regulatory Region (RR) in eukaryotic gene 2.

**Figure 1.**
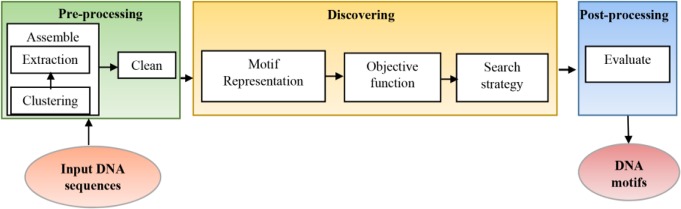
General block diagram of motif discovery technique.

Sequence motifs have constant size and are often repeated and conserved, but at the same time, they are tiny (about 6–12 *bp*) and the intergenic regions are very long and highly variable that make motif discovery a problematic task. These patterns play an essential role in recognizing Transcription Factor Binding Sites (TF-BSs) that help in learning the mechanisms for regulation of gene expression 3. Different types of motifs are planted motifs, structured motifs, sequence motifs, gapped motifs and network motifs 4. Motif discovery problem in a simple form can be formulated as in figure 1 where the input is DNA sequence with unknown motifs at different unknown positions with various lengths and the output is the DNA motifs. The motif discovery technique consists of three main stages 5:

### A. Pre-processing

It is preparing the DNA sequences for accurate motif discovery by assembling and clean steps. In assembling step, it is advised to select as many target sequences as possible that may contain motifs, try to keep sequences as short as possible, and remove sequences that are unlikely to contain any motifs. Assembling step is done by clustering the input sequences based on some information and then extracting the desired sequences in an appropriate sequence database. Then, cleaning the input sequences to mask or remove confounding sequences is necessary.

### B. Discovering

The middle stage is the motif discovery approach that begins by representing the sequences. There are two ways to represent the motifs: consensus string and Position-specific Weight Matrices (PWM). Consensus string has the same length of DNA sequence motif; it allows to degenerate symbols in a string using IUPAC code while PWM is a matrix of 4xm where m is the motif length. Every position in the matrix represents the probability of each nucleotide at each index position of the motif. After motif representation, the suitable objective function is determined and finally appropriate search algorithm is applied. There are hundreds of algorithms for motif extraction that most of them are listed in table 1.

**Table 1.** Motif discovery algorithms

**No.**	**Algorithm**	**Operating principle**	**Ref.**
**Enumerative approach**			
1	YMF		(29)
2	DREME	Simple word- based	(9)
3	oligonucleotide analysis		(30)
4	CisFinder		(10)
5	By Thomas *et al*	Simple word-based with Clustering technique	(31)
6	POSMO		(32)
7	Weeder		(7)
8	FMotif		(11)
9	By G. Pavesi		(33)
10	MITRA	Tree-based	(34)
11	CENSUS		(35)
12	RISOTTO		(36)
13	SLI-REST		(37)
14	DRIMust		(38)
15	MCES	Tree based with clustering technique	(12)
16	WINNOWER		(39)
17	Pruner		(40)
18	cWINNOWER		(41)
19	By Sze *et al*		(42)
20	RecMotif	Graph-theoretic	(43)
21	ListMotif		(44)
22	TreeMotif		(45)
23	GWM		(46)
24	GWM2		(47)
25	Voting		(48)
26	PMS1		(49)
27	PMS2		(49)
28	PMS3		(49)
29	By Sze *et al*		(50)
30	PMSi		(51)
31	PMSP	Fixed candidates	(51)
32	Stemming		(52)
33	PMS4		(53)
34	PMS5		(54)
35	PMS6		(55)
36	PairMotif		(56)
37	iTriplet		(57)
38	PMSPrune		(58)
39	Pampa		(59)
40	PMS3p		(60)
41	Provable	Modified candidate	(61)
42	qPMSPruneI		(62)
43	qPMS7		(62)
44	By Tanaka *et al*		(63)
45	Random projection		(64)
46	Uniform projection	Hashing	(65)
47	Low-dispersion projection		(66)
48	MULTIPROFILER	Extended sample-driven (ESD)	(67)
49	Pattern Branching		(68)
50	Ref Select	Reference selection	(69)
Probabilistic approach			
51	MEME		(14)
52	STEME	EM	(15)
53	EXTREME		(16)
54	Profile Branching		(68)
55	APMotif	EM with clustering	(70)
56	AlignACE		(17)
57	SPWDM		(71)
58	By Lawrence *et al*	Gibbs sampling	(72)
59	Motif- Sampler		(73)
60	BioProspector	Gibbs Sampling with hidden markov	(18)
62	MITSU		(74)
63	MCEMDA	Stochastic Expectation Maximization (sEM)	(75)
64	SEAM		(76)
65	By Jensen *et al*		(13)
66	LOGOS		(77)
67	BaMM		(78)
68	By Jääskinen *et al*		(79)
69	By Frith *et al*	Baysian approach	(80)
70	SBaSeTraM		(81)
71	By Wakefield *et al*		(82)
72	MotifCut		(83)
73	MCL-WMR	Graphic based	(84)
74	EPP	Entropy-based position projection	(6)
75	CONSENSUS	Greedy Algorithm	(85)
76	By Huang *et al*	heuristic algorithm	(86)
GA			
77	St-GA		(87)
78	GAMI		(88)
79	FMGA	Simple GA	(89)
80	MDGA		(90)
81	By Paul *et al*		(91)
82	By Vijayvargiya *et al*	Clustering	(92)
83	By Gutierrez *et al*		(93)
84	GARPS		(94)
85	GAEM		(95)
86	GADEM		(96)
87	CompareProspector		(97)
88	By Fatemeh *et al*	Hybrid	(98)
89	GEMFA		(99)
90	MRPGA		(100)
91	By Xiaochun *et al*		(101)
92	GAME		(19)
93	By Yetian *et al*		(102)
94	By Li *et al*	Others	(103)
95	MOGAMOD		(104)
PSO			
96	PMbPSO	Standard PSO	(4)
97	LPBS		(105)
98	PSOMF		(106)
99	Lei *et al*		(107)
100	Lei *et al*		(108)
101	DSAPSO	Modified PSO	(109)
102	By Karabulut *et al*		(110)
103	Lei *et al*		(111)
104	Hardin *et al*		(112)
105	GSA-PSO	Hybrid	(113)
106	SPSO-Lk		(114)
ABC algorithm			
107	Multiobjective ABC		(115)
108	MO-ABC/DE	ABC	(116)
109	Consensus ABC		(8)
ACO algorithm			
110	Machhi *et al*	ACO with Gibbs sampling	(117)
111	MFACO		(118)
112	Cheng *et al*	ACO with EM	(119)
CS algorithm			
113	MACS	CS	(120)
Combinatorial			
114	STGEMS	Enumerative and probalistic approaches	(121)
115	MDScan		(122)
116	MUSA	Probabilstic and machine learning approaches	(123)
117	EMD	Multiple algorithms	(124)
118	MobyDick	Dictionary	(125)
119	WordSpy		(126)

### C. Post-processing

Post-processing evaluates the resultant motifs. This paper presents a more general classification of the sequence motifs extraction methods. Most of them are mentioned with a comparison among them.

## Literature Review

There are two principal types of motif discovery algorithms; *i.e*. enumeration approach and probabilistic technique. Enumeration approach searches for consensus sequences; motifs are predicted based on the enumeration of words and computing word similarities so this approach is sometimes called the word enumeration approach to solve Panted (l, d) Motif Problem (PMP) with motif length (l) and a maximum number of mismatches (d). The algorithms based on the word enumeration approach exhaustively search the whole search space to determine which ones appear with pos-sible substitutions and therefore it typically locates the global optimum. However, this also means that they are exponential-time algorithms that require a long time to detect the larger l and inefficient for handling dozens of sequences, so they are only suitable for short motifs 6. Moreover, these algorithms require many parameters determined by the users such as motif length, the number of mismatches allowed, and a minimum number of sequences that the motif has to appear in 7.

The word enumeration approach can be accelerated by using specialized data structures such as suffix trees or parallel processing 8. Popular algorithms based on this approach are DREME 9, CisFinder 10, Weeder 7, FMotif 11, and MCES 12.

A second group is a probabilistic approach. It constructs a probabilistic model called position-Specific Weight Matrix (PSWM) or motif matrix that specifies a distribution of bases for each position in TFBS to distinguish motifs *vs*. non-motifs and it requires few search parameters 13. The most popular methods based on probabilistic approach are MEME 14, STEME 15, EXTREME 16, AlignACE 17, and BioProspector 18.

Recently, new algorithms inspired from nature are presented that solve complex and dynamic problems with appropriate time and optimal cost. These algorithms simulate the behavior of insects or other animals for problem-solving. Evolutionary algorithms can over-come the disadvantages of local search and synthesize local search and global search 19. Examples of evolutionary algorithms are: Genetic Algorithm (GA) 20, Genetic Programming (Special type of GA) 21, Differential Evolution (DE) 22, Evolution Strategy 23, Multimodal Optimization 24, Cuckoo-Search (CS) 25, Levy flight 26, Bacterial Colony Optimization 27, and Intelligent Water Drops algorithm 28.

Swarm intelligence is a special class of evolutionary algorithm including Particle Swarm Optimization (PSO) 111, Artificial Bee Colony (ABC) algorithm 127, and Ant Colony Optimization (ACO) algorithm 128.

The beauty of nature-inspired algorithms is that they provide flexibility in evaluating the solutions by using fitness functions that score the solutions. These functions vary from problem to another and evaluate using different information types as biological information, functional information, *etc*. Moreover, these algorithms provide flexibility in motif representation 129.

**Figure 2.**
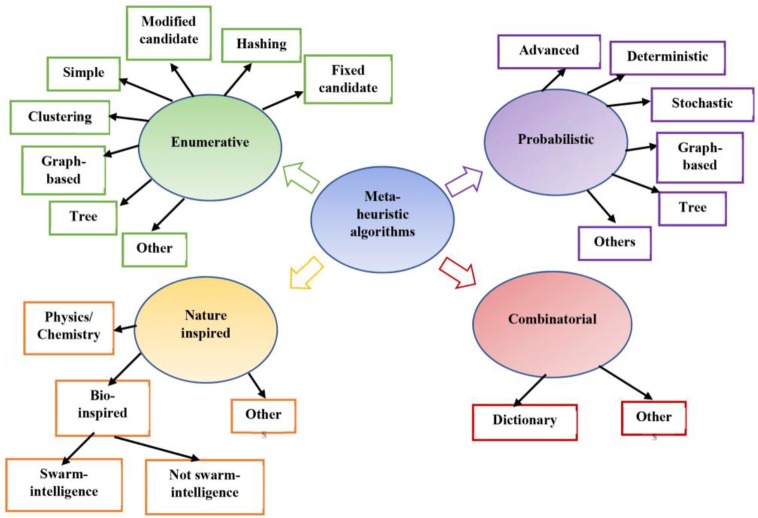
Classification of motif discovery algorithms as enumerative, probabilistic, nature inspired and combinatorial types.

Finally, the last category is a combinatorial algorithm that mixes multiple algorithms. The classification of motif discovery algorithms is shown in figure 2. This paper presents a classification of motif discovery algorithms and gives an overview of the most common algorithms with many examples; also, the main features while designing a new algorithm and future work are proposed.

### A. Enumerative approach

The enumerative approach can be classified into many classes.

### 1. Simple word enumeration

The first class is based on simple word enumeration. Some existing algorithms in this class are YMF 134 and DREME 9,29. Sinha *et al* 29 developed YMF (Yeast Motif Finder) algorithm that detects short motifs with a small number of degenerate positions in yeast genomes using consensus representation. YMF enumerates all motifs in the search space approach and calculates the z-score to produce those motifs with greatest z-scores. Bailey *et al* 9 proposed DREME (Discriminative Regular Expression Motif Elicitation) algorithm that also calculates the significance of motifs using Fisher’s Exact test. The algorithm starts with generating a set of short k-mers, followed by applying Fisher’s Exact test on two sets of DNA sequences (Input set and background set) using a significance threshold to calculate the significance of each word (No wildcards) of length three to eight that occurs in the positive sequences and select the 100 most significant words for being used in the inner loop where they passed as “seed” REs to perform a beam search that determines the most significant generalizations of them (One wildcard). To find multiple, non-redundant motifs in a set of sequences, outer loop determines the most significant motif using the heuristic search of RE motifs and the best motif found replaces its occurrences by a special letter; then the search process is repeated again many times until E-value of the new motif is less than the determined significance threshold.

### 2. Clustering-based method

Instead of using two loops for finding multiple motifs, the second class was proposed. Sharov *et al* 10 proposed word clustering method called CisFinder to detect short motif with high processing speed in large sequences (up to 50 *Mb*). Firstly, one should define nucleotide substitution matrix for each n-mer word, then calculate Position Frequency Matrices (PFMs) for n-mer word counts with and without gaps in both test and control sets. To generate non-redundant motifs, PFMs are extended over flanking and gap regions followed by means of clustering. Thomas *et al* 31 extended the CisFinder technique to deal with whole ChIP-seq peak data sets.

### 3. Tree-based method

The third class is a tree -based search to accelerate the word enumeration technique. Pavesi *et al* 7 presented Weeder algorithm based on count matching patterns with specific and most extreme mismatches. At first, the motifs are represented using consensus sequence and based on the difference between the k-mers of the input sequences and the consensus under a limited number of substitutions, k-mers are assembled and each group is evaluated with a specific measure of significance. The constructed suffix tree was proposed in FMotif 11 algorithm for finding long (l, d) motifs in large DNA sequences under the ZOMOPS (Zero, one or multiple occurrence(s) of the motif instance(s) per sequence) constraints. This proposed tree is faster than standard suffix tree as it avoids a large number of repeated scans of sequences on the suffix tree. Afterward, Qiang *et al* 12 proposed MCES algorithm for a PMP that used both suffix tree and parallel processing to deal with large datasets. MCES algorithm starts with mining step that constructs the Suffix Array (SA) and the Longest Common Prefix array (LCP) for the input datasets. At that point, combining step clusters substrings of various lengths to get predicted motifs.

### 4. Graph theoretic-based method

The graph-theoretic method represents a motif instance, as a clique; the graph G is built by representing each l-mer in the input sequences by vertex and the edge between a pair of vertices representing a pair of l-mer in different input sequences having the Hamming distance between the substrings which is less than or equal to 2d. Then, cliques of size N are searched for in this graph. Popular graph-theoretic methods are WINNOWER 39, Pruner 40, and cWINNOWER 41.

### 5. Hashing-based method

Buhler *et al* 64 developed random projection algorithm for a PMP that projects every l-mer in the input data into a smaller space by hashing. Initially, a projection of l-dimensional space onto a k-dimensional subspace for all subsequences in the input set is developed, and random projection is constructed by choosing random k positions from l position. Using this projection, each l-mer is hashed to its corresponding bucket. After projections, each bucket contains l-mer more than a threshold and this is called qualified bucket. Random hashing is repeated n times to ensure the qualified bucket at least more than once. Finally, profile for each of them should be computed to get the most probable l-mer in the sequence that was represented as consensus sequences. In previous studies, random projection was developed using uniform projection and low-dispersion projection algorithms, respectively 65,66.

### 6. Fixed candidates and modified candidate-based methods

The sixth class is fixed candidates that select candidate motifs from input sequences and use them for motif scanning while the seventh class is modified candidate that selects one candidate from the input sequence and modifies it letter by letter.

Finally, there is a proposed algorithm called RefSelect 69 to select reference sequences for PMP. The reference sequences are the sequences that don’t contain motif instances, so, this method tries to select the reference sequences that generate a small number of candidate motifs as possible. The algorithm consists of two steps; firstly, for every two sequences in input dataset D, the number of candidate motifs generated from them should be computed using the Hamming distance between every two l-mers. Then, the set with candidate motifs, as small as possible, is selected as a reference set.

### B. Probabilistic approach

***Deterministic approach:*** Expectation-Maximization (EM) 130 is the famous example of deterministic approach. EM for motif finding was first introduced by Lawrence *et al* 132 and it consists of two main steps, the first called “Expectation step” that estimates the values of some set of unknowns based on a set of parameters. The second step is “Maximization step” that uses those estimated values to refine the parameters over several iterations. EM is used to identify conserved areas in unaligned DNA and proteins with an assumption that each sequence must contain one common site, the parameters; in this case, they are the entries in the PWM and the background nucleotide probabilities while our unknowns are the scores for each possible motif position in all of the sequences.

There are several algorithms based on EM. MEME (Multiple EM for Motif Elicitation) 14 is a popular motif recognition program that optimizes PWMs using the EM algorithm. It has several versions 132–136. The idea of MEME algorithm is to find an initial motif and then use expectation and maximization steps to improve the motif until the values in the PWM do not improve or the maximum number of iterations is reached. The MEME algorithm starts from a single site, *i.e*. k-mer (Random or specified) and estimates motif model (PWM). For every possible location in every input sequence, the probability, given the PWM model, should be identified to detect examples of the model; then, the motif model should be re-estimated by calculating a new PWM. EM alternates between examples of the model step and re-estimates the motif model step. A single iteration for each k-mer in target sequences should be performed and the best motif from this site needs to be selected and then only the one to converge should be iterated. The algorithm searches for new motifs after erasing the old discovered motif. It defines all three types of motif discovery sequence model: OOPS, ZOOPS, and TCMs corresponding to one occurrence per sequence, zero or one occurrence per sequence, and zero or more occurrences per sequence, respectively. Reid *et al* 15 presented STEME (Suffix Tree EM for Motif Elicitation) algorithm to accelerate the MEME algorithm and the first application of suffix trees to EM algorithm was considered. Quang *et al* 16 also tried to accelerate the MEME algorithm by developing EXTREME (Online EM algorithm for motif discovery) algorithm. EXTREME is an online web server that implements the MEME algorithm and can work on a large dataset without discarding any sequences.

### 2. Stochastic approach

Gibbs sampling 72 is a famous stochastic approach, similar to EM algorithm. Pseudocode of the Gibbs sampling algorithm for motif detection follows these steps 130:

- Random initializing of motif positions in the input N sequences with an assumption of the presence of one motif per sequence,
- Choosing one sequence at random,
- Computing PWM for the other N-1 sequences using staring positions of motifs and background probabilities for each base using the non-motif positions,
- Calculating probability of each possible motif location in the removed sequence using PWM and background probabilities,
- For the removed sequence, choosing a new starting position based on step 4.

Steps 2–5 should be iterated until the values in the PWM do not improve or the maximum number of iterations has been reached.

Many methods 17,18 have been developed that implement the concept of Gibbs’ sampling to extend its functionality. Hughes *et al* 17 proposed Align ACE (Aligns Nucleic Acid Conserved Elements) algorithm based on Gibbs’ sampling with some improvements: (1) The motif model was changed to fit the source genome because the base frequencies for non-site sequence is fixed, (2) Both strands of the input sequence are considered and no circumstance overlapping is allowed, (3) Iteratively, aligned sites were masked out to find multiple different motifs, and (4) It uses an improved near-optimum sampling method.

BioProspector 18 algorithm is also based on Gibbs’ sampling with several improvements: (1) It uses a Mar-kov model estimated from all promoter noncoding sequences to represent the non-motif background in order to improve the motif specificity, (2) It can find two-block motifs with variable gap, and (3) Sampling with two thresholds allows every input sequence to include zero or multiple copies of the motif.

### 3. Advanced approach

Different algorithms were proposed based on Bayesian approach 137. Jensen *et al* 13 proposed an algorithm based on Bayesian approach with Markov chain Monte Carlo. Xing *et al* 77 proposed LOGOS (Integrated LOcal and GlObal motif sequence model) algorithm that combines between HMDM (Hidden Markov Dirichlet-Multinomial) for local alignment model for each different motif and HMM (Hidden Markov model) for global motif distribution model for the occurrence of multiple motifs.

Recently, Siebert *et al* 78 developed a Bayesian Markov Model (BaMM) approach that trains higher order Markov models to build the dependency model. BaMM algorithm is more complex than PWMs wherein the PWMs cannot model correlations among nucleotides because PWMs nucleotide probabilities are independent of nucleotides at other positions. In the proposed algorithm, Bayesian approach using Markov models makes optimal use of the available information while avoids training by the decrease in number of parameters.

Other different algorithms 79,80 were presented like clustering methods based on Bayesian approach.

### 4. Others

EPP (Entropy-based position projection) 6 algorithm was proposed to escape from local optima. This algorithm based on projection process depends on the relative entropy in each position of motif instead of random projection.

### C. Nature-inspired algorithms

Nature-inspired algorithms are classified according to the sources of inspiration into three main categories 138. They are swarm-intelligence, non swarm-intelligence and physics/chemistry as shown in figure 2. Popular evolutionary algorithms used in motif discovery are GA and PSO and few of them are ABC, CS, and ACO.

### 1. GA

GA is a probabilistic optimization algorithm based on evolutionary computing. GA is inspired from biological evolution processes like selection, crossover, and mutation. The motivation for using GA comes from the idea of reducing the number of searches in a high number of DNA sequences. The basic structure of GA consists of a population of candidate solutions throughout several generations to find the best solution or set of possible solutions. The algorithm starts with the random generation of individuals that are then evaluated by a fitness function. At that point, a selection process selects new individuals called offspring that have some features of the parents and the others are discarded, then the genetic operators are applied on offspring. The parents are selected based on a probabilistic process biased by their fitness value using specified selection techniques that keep the diversity of the population large and prevent premature convergence on poor solutions. The most common technique for parent selection is the roulette-wheel method. In a roulette wheel selection, based on the number of individual say n, the circular wheel is divided into n pies. The size of each pie is proportional to the fitness of the element. The roulette wheel is spun randomly and the element where it stops is chosen as the parent. The selected two individuals are used to produce offspring; this means the elements of higher chance for selection have higher fitness.

After two selections of parents, a crossover is applied to select a random site, and the rightmost strings are swapped to produce new children, followed by applying the mutation process by changing the value of some selected position. The process is iteratively repeated until some stop criterion is reached on the satisfactory fitness level 139. Some methods 89,90 use standard GA. Liu *et al* 89 introduced FMGA algorithm that is based on simple GA. The genetic operators were specialized for motif discovery problem; the mutation operator was applied using PWM to reserve the completely conserved positions and the crossover operator was implemented with specially-designed gap penalties that optimize a scoring function. Che *et al* 90 proposed MDGA algorithm that is also based on simple GA. Other methods were proposed and most of them 91–93 used population clustering technique that partitions population into subpopulations before mating. Clustering is done by the similarity between solutions using data clustering algorithms.

Using population clustering technique, Paul *et al* 91 proposed an algorithm for PMP to detect multiple and weak motifs. Firstly, population initialization by random selecting of subsequences of motif length used to form a candidate consensus motif is done and then all input sequences are scanned to detect all similar substrings followed by sorting them according to a number of mismatches of each substring from the candidate motif. Next, scoring function is applied on them. This method was extended to detect weak motifs using alignment score metric and clustering technique. Finally, fitness of an individual (Cluster) is calculated to select the parents for using in GA. Vijayvargiya *et al* 92 proposed an algorithm with the position based representations of individual and clustering of population scheme. Gutierrez *et al* 93 proposed a new statistical GA that mixes GA structure with several statistical coefficients. In this method, all the input sequences are joined in a single super-sequence and the individuals are represented by a single position value; at that point, the super-sequence is separated into subsequences of any length regardless of the length of each sequence. For each generation of the population, the fitness value will be calculated against one of the subsequences. The fitness function is a combination of different functions applied at different moments of the process. The algorithm is started by checking if they are overrepresented in the given subsequence by calculating the difference of similar words between the candidate motif and the background motif. Then, the selection process is applied consisting of two steps; the first is using the Fluffiness Coefficient by the mean and standard deviation of the simple overrepresentation values and the individuals with the lowest Fluffiness value are eliminated from the population. The second step is using different coefficients to decide if the candidates are the final solutions of the problem or not for an individual has survived for at least 10 generations. The first coefficient is Thinness coefficient where the individuals with a Thinness coefficient above 0.6 are eliminated from the population. Mann-Whitney is the second coefficient for two populations to quantify, if one of them has a tendency to have larger values than the other. The first sample corresponds to the vector with the values stored for the number of similar words for the candidate motif in each generation, and the second sample is formed by the same values for the background motif. If the probability of both data samples coming from the same population is lower than 0.05, at that point, the motif is considered as a possible final solution. The third step is the creation of offspring by applying genetic operators, one-point crossover, and random mutation, on the selected parents. Finally, filtering and clustering of solutions is another method for every given motif width to generate the final solutions.

Some methods enhance the GA by using hybrid methods that combine GA with another technique 94–96,140 or propose additional operators in addition to basic genetic operators 19,102.

Huo *et al* 94 proposed a new algorithm (GARP) that optimizes GA based on the random projection strategy (RPS) to identify planted (l, d) motifs. The idea behind using RPS before GA is to find good starting positions for being used in simple GA as an initial population instead of random population. Wang *et al* 95 presented GAEM algorithm that combines GA and EM for planted edited (l, d)-motif finding problem. In planted edited (l, d) -motif finding problem, the mutation in motifs due to substitution, deletion, and insertion, causes the motifs to have different lengths ranging from l-d to l+d. The EM algorithm is used after the random initial population to get the best starting positions to be used as a seed to GA. Wei *et al* 19 introduced GAME (Genetic Algorithm for Motif Elicitation) that proposes two operators called ADJUST and SHIFT to escape from local optima. The ADJUST operator escapes from a local optimum that occurs if any of the motif sites has not been aligned correctly by checking every possible site position in a sequence and choosing the best match to the sites in the other sequences. But SHIFT operator escapes from local optima that occur when all motif sites are slightly misaligned by shifting the subsequences in the direction which gives the best fitness. Yetian *et al* 102 also presented a new operator called addition to the standard GA operators. The algorithm started with a motif length of three and then the proposed operator is used until the length of the optimal motif reaches to the standard level. The result achieves a higher score than the other three methods: Gibbs Sampler 141, GA 142 and GARPS 94 algorithm.

### 2. PSO

PSO is a new global optimization technique 143 for solving continuous optimization problems. PSO algorithm is characterized by its simple computations and information sharing within the algorithm. It simulates the social behaviors of organisms movements in flocks of birds or schools of fish to find food sources and defenses against predators. Each particle uses its own flying experience and flying experience of other particles to adjust its “flying” so it combines self-experiences with social experiences. In self-experiences, the particle tries to get local best particle position and this is done by the particle stores. The best solution visited so far in its memory is called pbest, and it has an attraction towards this solution as it navigates through the solution search space. Social experiences are utilized to get best global particle position through the particle stores and the best solution visited by any particle and attraction towards this solution is called gbest. For an n-dimensional search space, the position and velocity of the ith particle are represented by Yi= (yi1, yi2, …, yin) and Vi= (vi1, vi2, vi3 …, vin), respectively. The previous best position is denoted as Pi= (pi1, pi2, …, pin). Pg is the global best particle in the swarm. For the swarm S, the new velocity of each particle is calculated according to the following equation:

Vin (t+1) = vin(t)+c1 r1 (pin−yin) +c2 r2 (pg−yin)

(1) The position is updated using:

Yin (t+1) = Yin (t)+ Vin (t+1)

(2) Where i = 1, 2… S represents the particle index and n=1, 2… N represents the dimension. c1 and c2 are cognitive and social scaling parameters, respectively.

At each iteration, pbest and gbest are updated for every particle as per their fitness values. The procedure is iteratively repeated until some stop criterion is re-ached or satisfactory fitness level has been reached.

PSO has wide applications and has been proven to be effective in motif finding problems 112. In recent years, there are few numbers of researches which utilized PSO to solve different types of motif finding problems. Chang *et al* 144 proposed a modified PSO algorithm where the position and velocity are adjusted to escape from local optima. Hardin *et al* 112 proposed a hybrid motif discovery approach based upon a combination of PSO and EM algorithm and PSO was utilized to generate a seed for the EM algorithm. In previous studies, modified PSO algorithm was proposed based on word dissimilarity graph 107,108. Modified PSO algorithm began to break all input sequences into l-mers and develop a novel mapping scheme that was used in the fitness function. Each particle kept track of a vector of locations in each given sequence and formed a consensus sequence. Then, the updated policy of PSO was modified where the new and current motif positions must be in the upper and lower bounds of the velocity. To escape from local optima, the algorithms scan all input sequences after gbest value reaches to a certain threshold to check if l-mer has a fitness value in comparison to gbest. Also, the method used “reset move” that moves all the current solution, pbest and gbest by a random distance. Finally, the termination criteria are determined automatically using repeat -based method. Reddy *et al* 4 developed PMbPSO (PSO-based algorithm for Planted Motif Finding) algorithm. PMbPSO algorithm selects initial positions for all motifs by random and generates ten children for each parent (Motif) and then computes the fitness function for each parent and its children to get the best position; at that point, the best position from all particles is got followed by updating velocity and position for each particle for a number of iterations. Haruna *et al* 105 integrate Linear-PSO with binary search technique (LPBS) to minimize the execution time and increase the validity in motif discovery of DNA sequence for specific species. LPBS algorithm starts with initializing the population by selecting the target motif from the reference set and searching for similar motifs using the binary search, then applying the standard PSO algorithm to discover motifs. Recently, Ebtehal *et al* 113 introduced a hybrid GSA-PSO algorithm that combines local search capabilities of the Gravitational Search Algorithm (GSA) and global search capabilities of PSO algorithm.

### 3. ABC algorithm

ABC algorithm is a type of swarm-based algorithm proposed by Karaboga 145. It simulates the behavior of honey bees to find a food source. Two fundamental properties to obtain swarm intelligent behavior in honey bee colonies are self-organizing and division of labor. The bee colony contains two main groups which are employed and unemployed foragers. Employed bees are going to the food source which is visited previously and they are responsible for giving information to unemployed foragers about the quality of the assigned nectar supply. Many factors determine the value of a food source like its distance from the nest, its energy concentration and the degree of difficulty to extract this energy. Unemployed bees are categorized to scout and onlooker bees. Scout bee searches around the nest randomly to find new food sources while onlooker bee uses the information shared by employed foragers to establish a food source.

Employed bees’ numbers are the same for food sources’ numbers around the hive. At first, scout bees initialize all positions of food sources that represent possible solutions to the problem. Then, employed bees and onlooker bees exploit the nectar of food sources that corresponds to the quality (Fitness) of the associated solution, and this continual exploitation will finally cause them to become exhausted. The employed bee which turned into exploiting the exhausted food source turns into a scout bee looking for other food sources. The ABC algorithm involves four fundamental phases: A neighbor food source FS is determined and calculated by the following equation:

$$FS_{i}-f_{i}+\text{rand}\;(-1,\;1)*(f_{i}-f_{k})\;(3)$$

Where i is a randomly selected parameter index, *f_k_* is a randomly selected food source, rand (−1, 1) is a random number between −1, 1.

The fitness of this new food source is calculated and a greedy selection is applied between it and its parent *i.e*. between *FS* and *F.* From that point, employed bees share information about their food source via dancing on the dancing area with onlooker bees waiting inside the hive.

Gonzalez-Alvarez *et al* 115 developed multiobjective ABC algorithm that aims to adapt the ABC algorithm to multiobjective context. The multiobjective optimizes more than one objective function at the same time to get a set of optimal solutions known as Pareto set. This algorithm defines three conflicting objectives as motif length, support, and similarity and multiobjective adaptations of ABC including multi-term fitness function, ranking, and sorting methodology are used. González *et al* 116 proposed Multiobjective Artificial Bee Colony with Differential Evolution algorithm (MO-ABC/DE) that combines the general schema of ABC with Differential Evolution. Recently, Karaboga *et al* 8 proposed consensus ABC algorithm. This is a discrete model based on a similarity value between consensus sequences of the ABC algorithm. The algorithm starts with random initialization, and then calculates the similarity value of the resultant consensus sequence followed by a new neighborhood selection method that is based totally on the similarity values of the consensus sequences.

### 4. ACO algorithm

The ACO algorithm 128 is a metaheuristic optimization technique that mimics the behavior of real ants, which try to find the shortest path to the food from their nest. The ants explore randomly the area surrounding their nest, and while moving, they leave a chemical pheromone trail on the ground that helps them to go to the nest. Ants interact with each other through this chemical component. The quantity of pheromone is proportional to the quantity and the quality of the food and this pheromone will be guided to other ants for the food source. When evaporation occurs, it reduces the attractive strength of pheromone. The evaporation takes a long time in the shorter path than the longest. The ants choose their way with strong pheromone concentrations. The characteristics of ACO algorithm are: (1) It depends on two variables including the amount and the evaporation of pheromone. The amount of pheromone is directly proportional to the richness of the food and inversely to evaporation; evaporation factor avoids the convergence to a locally optimal solution, (2) Ants act concurrently and independently, and (3) The behavior is stigmergy *i.e*. the interaction between ants is indirect, (4) Ants can explore vast areas without global view of the ground, (5) Starting point is selected at random.

The pheromone probability in terms of motif discovery is given by:

(4)
$$P_{a}(\mathrm{lc})=\frac{{\left(\omega_{lc}(t)\right)}^{\alpha }\left(\eta_{lc}^{\beta }\right)}{\sum_{u\in \left\{A,T,C,G\right\}}{\left(\omega_{lu}(t)\right)}^{\alpha }(\eta_{lu}^{\beta })}$$

Where *P_a_ (ic)* is the probability of ant a choosing c in position l, ƞ_ic_ is the heuristic information to measure the frequency of letter c in input sequences, i.e. the weight of the letter c, β and α represent the influence of the pheromone trails, c is the character set of input sequences, ω_ic_ (t) is the amount of pheromone on the character c at position l at time t, and ω_iu_ (t) is the amount of pheromone on neighborhood at position l which ant a has not visited yet at time t. An updated pheromone trial is:

(5)
$$\omega_{\mathrm{lc}}(t+1)\;=(1-\rho ).\omega_{\mathrm{lc}}(t)+\sum_{k=1}^{y_{\mathrm{lc}}}\Delta w_{\mathrm{lc}}^{k}(t)$$

Where y_ic_ is the total number of ants, which carry the character c at position l, p is the rate of the pheromone trails evaporation (0<p<1), and $\Delta w_{\mathrm{lc}}^{k}$ is the variable of pheromone deposited by kth ant on the character c at position l. A lot of methods used ACO algorithm in motif discovery. Machhi *et al* 109 used ACO algorithm with a Gibbs sampling algorithm. An ACO algorithm finds better starting positions of the sequences provided as starting position for the Gibbs sampler method instead of random initialization. This algorithm starts with each ant choosing the path to construct a sample with motif length (m) and that depends on pheromone probability. Then, each ant is compared between the selected sample (m) and each substring in input sequences to get the set that represents the best matching substrings. Next, fitness function for each selected set is calculated. After that, the amount of pheromone is updated and finally iterated until no change.

### 5. CS algorithm

CS is a new simple heuristic search algorithm that is more efficient than GA and PSO 146–148. CS is inspired from brood parasitism reproduction behavior of some cuckoo species in combination with Lévy flight behavior 149. The cuckoos lay their eggs in nests of the other birds with the abilities of selecting the lately spawned nests and removing existing eggs to increase the hatching probability of their eggs. If host birds discover these eggs, they either throw them away or abandon the nest and build a new nest. CS is characterized by the subsequent rules:

Each cuckoo lays one egg at a time and disposes its egg in a random selected nest.

The nests that have high quality of eggs (Solutions) are the best and will continue to the following generations.

The number of accessible host nests is fixed, and a host bird can discover a parasitic egg with a probability Pa E [0,1].

To simulate the behavior of cuckoo reproduction, each egg in a nest is a solution and each cuckoo’s egg is a new solution. The aim is to supplant a not-so good solution in the nests with newer and better solutions by Lévy flights:

(6)
$$y_{i}^{\left(t+1\right)}=y_{i}^{t}+\alpha ⊕\mathrm{Levy}(\lambda )$$

Where $y_{i}^{\left(t+1\right)}$ is a new solution, $y_{i}^{t}$ is the current location, α is the step size and Levy(λ) is the transition probability or random walk based on the Lévy flights. CS is an effective global optimization algorithm and has many applications in different fields 150. Ebtehal *et al* 120 applied CS and Modified Adaptive Cuckoo Search (MACS) algorithm on PMP. The MACS algorithm enhances the basic CS algorithm by grouping parallel, incentive, information and adaptive strategies.

## DNA Motif Databases

### Synthetic DNA sequences

The simulated data set was first introduced by Pevzner et al 39 to create planted (l, d) motif in n sequences selected randomly from a set of N sequences where n≤N. There are online sites 151,152 to automatically create planted (l, d) motif.

### Real DNA sequences

There are several online databases of DNA motifs listed in table 2 with a short description of each one.

**Table 2.** Real datasets of DNA motifs

**Database**	**Link**	**Description**
**TRANSFAC**	http://gene-regulation.com/pub/databases.html	TRANSFAC is the database of eukaryotic TFs, their genomic binding sites, and DNA-binding profiles
**JASPAR**	http://jaspar.genereg.net/	A public dataset of motifs for multicellular eukaryotes
**PROSITE**	http://prosite.expasy.org/	PROSITE includes documentation sections describing protein domains, families and functional sites in addition to related patterns and profiles to recognize them
**YEASTRACT**	http://www.yeastract.com/	It contains predicted TFs for S. cerevisiae.
**SCPD**	http://rulai.cshl.edu/SCPD/	
**RegulonDB**	http://regulondb.ccg.unam.mx/	Provides curated information on the transcriptional regulatory network of E. coli and contains both computational as well as experimental data of predicted objects
**CisBP**	http://cisbp.ccbr.utoronto.ca/	It contains a list of >160,000 predicted TFs from >300 species
**DBTBS**	http://dbtbs.hgc.jp/	It contains TFs for Bacillus subtilis

## Discussion

As sequencing technology has improved, the volume of biological sequence data in public databases increases and this increases the importance of motif discovery in computer science and molecular biology 153. Motif discovery has some difficulties: (1) Motifs are not identical to each other, (2) The motif sequence is unknown, (3) Motif location is unknown, (4) Existing of random motifs, and (5) The location of a motif in each sequence is unrelated to other ones.

The motif discovery algorithms are classified into two major groups as enumerative approach and probabilistic approach. As the name of the first class imparts, it is counting and comparing oligonucleotide frequencies for all possible motifs, based on specific motif model description. It has some advantages: (1) Global optimality, (2) It is appropriate for short motifs; therefore, it is useful for motif finding in eukaryotic genomes, (3) With optimized data structures, it becomes fast, and (4) Can find totally constrained motifs. However, the problems of this approach are: (1) It needs to be post-processed with some clustering systems as the typical transcription factor motifs often have several weak constrained positions, (2) It suffers from the problem of producing too many spurious motifs, (3) It represents certain numbers of motifs (11 wild cards), and (4) Long time processing is another problem as it checks every possible substring in the input dataset.

There are many algorithms based on sub-categories of this approach. The first sub-category is based on the enumerative approach without any enhancement; this leads to getting all possible motifs but at the same time there are many disadvantages which were mentioned above. YMF is designed for yeast genomes, it can’t detect motifs with large length or the number of degenerate positions is significant. DREME is a discriminative motif discovery tool to discover multiple, short, non-redundant, statistically significant motifs in short runtime using simplified form of regular expression words (11 wildcard characters). DREME algorithm was tested on ChIP-seq datasets [13 mouse ES Cell (mESC), 3 mouse erythrocytes and one human cell line (ChIP-seq datasets)]. DREME is compared to MEME algorithm and the results show that DREME algorithm can correctly predict motifs on ChIPseq experiment sequences in a shorter runtime than MEME. However, it can only find short motifs (from 4 to 8 *bp*) and an occurrence of the motif is well defined, as is the number of possible motifs of a given width.

The second sub-category is based on simple enumerative approach but it can discover multiple and weak motifs at the same time so, it is considered as the small enhancement of simple-based method. CisFinder technique tested on ChIP-seq data of TFs was expressed in ES cells. CisFinder can accurately identify PFMs of TF binding motifs and it is faster than MEME 133, WeederH 154, and RSAT 31. CisFinder can find motifs with a low level of enrichment, but it does not support outputting motifs of a specified length.

The algorithm proposed is similar to CisFinder algorithm, but it supports outputting motifs of a specified length 31. They reported that this algorithm combined accuracy and low computational time, but it is limited to short (l, d) motifs.

The first two sub-categories have nearly the same disadvantages, but they are time-consuming. There are many suggestions to improve this problem; in tree-based methods, it is also based on enumerating all possible motifs but it is time-consuming using suffix tree. Weeder algorithm accelerates the word enumeration technique by using a suffix tree, but it operates with a low efficiency for long motifs 11. FMotif algorithm could identify unknown motif lengths on ChIP-enriched regions. Next, MCES algorithm is a more powerful algorithm and there are two contributions in the miming step; it uses an adaptive frequency threshold for each possible length and it is based on Map Reduce strategy to deal well with very large datasets. The MCES is tested on simulated data and real data (ChIP-seq) and the results show that MCES can find the motif like the published one and run in a short time. MCES has many advantages like identifying motifs without OOPS constraint, handling very large data sets, handling the full-size input sequences and making full use of the information contained in the sequences, completing the computation with a good time performance and good identification accuracy.

Graph-based techniques are the same simple-based techniques but they represent the motif-instance by a graph to facilitate the search strategy.

There are many search strategies to enhance time operator.

Sub categories from five to seven try to enhance time operator using random concept whether random searching about the motif like hashing strategy or selecting random candidate motif or many candidate motifs.

However, the random projection algorithm takes long time operations as it depends on random initialization and it repeats the process for n times.

In fixed candidates and modified candidate-based techniques, the technique scans all input sequences to get the matched motifs.

In the probabilistic approach, the probability of each nucleotide base to be present in that position of the sequence is multiplied to yield the probability of the sequence. PWM is an appealing model due to its simplicity and wide application and it can represent an infinite number of motifs 15 but it has some problems 155: (1) It scales poorly with dataset size, (2) PWM representation assumes the independence of each position within a binding site, while this may be not true in reality, and (3) It converges to locally optimal solution.

EM algorithm is a popular example of probabilistic approach, but it has some limitations: (1) It converges to a local maximum, (2) It is extremely sensitive to initial conditions, (3) It assumes one motif per sequence, and (4) The running time of EM is linear with the length of the input sequences. MEME added several extensions to overcome these limitations where MEME runs the EM algorithm many times from different starting points using every existing l-mer in the sequence dataset. However, the running time scales poorly with the size of the dataset so, MEME algorithm can discover motifs in a satisfactory time by a compromising strategy such as discarding a majority of the sequences but discarding data is far from ideal as it can decrease the chance of discovering motifs corresponding to infrequent cofactors 156. STEME runs faster than the MEME algorithm, but with a large dataset, it finds motifs up to width 8 as its efficiency decreases quickly as the motif width increases. EXTREME achieves better running time, but at the same time, it requires too much storage space for processing large data. Gibbs sampling algorithm is another example of a probabilistic approach. It converges to local optimum, and is less dependent on initial parameters, but more dependent on all sequences exhibiting the motif. Advanced methods based on Bayesian technique are a subclass of probabilistic approach; examples of this class are the speedy algorithms with better objective function and BaMM algorithm 13. BaMM was tested on 446 human ChIP-seq datasets and the results show that the precision increases by 30–40% compared to PWM. However, BaMM algorithm has some limitation: (1) It uses EM algorithm which has some disadvantages as described above, (2) The running time is longer than EM algorithm which makes it difficult to apply on a big data set, (3) It defines ZOOPS model only, and (4) It requires motif length as an input parameter. Finally, EPP algorithm can be applied to OOPS, ZOOPS, and TCM sequence models and the results indicate that it can efficiently and effectively recognize motifs.

Evolutionary algorithms have been recognized due to their advantages of synthesizing local search and global search 94. GA and PSO are the famous evolutionary algorithms. GA is a discrete technique, but PSO is a continuous technique that must be modified to handle discrete design variables. GA and PSO are similar in the presence of interaction between the population’ members, but unlike GA that changes the population from generation to another, PSO keeps the same population; moreover, PSO does not have genetic operators and no notion of the “survival of the fittest”.

Based on GA, a lot of algorithms are proposed. The simple GA favors selection of the fittest, which may be a biologically meaningless solution and this tends to remove the diversity of the population. Simple GA identifies OOPS model only and ignores ZOOPS and TCM models which are not correct because some sequences contain multiple or other weak motifs that also need to be identified 157. FMGA and MDGA algorithms are examples on simple GA. FMGA performs better than MEME and Gibbs sampler algorithms. MDGA algorithm is compared with a Gibbs sampling algorithm when tested on real datasets 158 and the results showed that it achieves higher accuracy in short computation time; the computation time does not explicitly depend on the sequence length. To overcome simple GA problems, clustering approach was presented. Clustering scheme enables to retain the diversity of population over the generations and it can find various motifs. Based on clustering technique, a new scoring function was developed that takes some consideration like, the number of mutations, and the number of motifs per sequence 91. The authors reported that it gives effective result when it’s applied to simulated and real data. Previous methods presented by Vijayvargiya *et al* 83 could identify multiple motifs of the same length and discover long motifs when tested on synthetic and real datasets 92. Gutierrez *et al* 93 presented a new algorithm that has many advantages; there are no assumptions about the presence of the motifs in the input sequences, it is a heuristic algorithm, variable-length gaps can be predicted, and the background set of sequences is generated by shuffling the candidate motifs instead of shuffling the sequences themselves. The algorithm was tested on 52 data sets of four different organisms and the efficiency was compared with 14 methods using eight statistics. The proposed method gives its best results with fly and mouse data sets. However, when connecting all input sequences into one sequence and then dividing it, it may cause loss of motifs. Based on hybrid methods, GARP algorithm is better than the projection algorithm when tested on both simulated and real biological data, while GAEM algorithm tested on a simulated dataset, gives a success rate higher than HIGEDA 140 and when tested on a real dataset, it performs better than HIGEDA, GLAM2, and MEME. Finally, based on algorithms that enhance genetic operators, the GAME algorithm performs better than MEME and BioProspector when applied on simulation and real-data; the algorithm presented by Fan *et al* 102 has a higher score than the other three methods of Gibbs Sampler 141, GA 142, and GAR-PS 94.

From the proposed algorithms based on GA, it can be concluded that the methods presented previously 19,90,92,94,102 used simple fitness function although there are a lot of suggestions to improve this function. Though the methods proposed by Paul *et al*, Vijayvargiya *et al*, and Gutierrez *et al* 91–93 can identify ZOOPS model, they cannot identify multiple motifs of variable lengths instead of multiple motifs of the same lengths. Methods by Paul *et al* and Vijayvargiya *et al* 91,92 ignore the TCM model, while a method by Gutierrez *et al* 93 identifies it. The methods of Paul *et al*, Vijayvargiya *et al*, and Gutierrez *et al* 91,93 enhance the selection strategy by proposing new fitness function. The methods proposed by Huo *et al* and Wang *et al* 94,95 enhance the GA algorithm by combining it with another algorithm to get the starting positions to be used as seed to the GA algorithm, but this increases the computational time also. The selection of EM in the method by Wang *et al* 95 is a bad choice as EM has many limitations as mentioned above. The proposed methods by Wei *et al* and Fan *et al* 19,102 try to enhance the GA algorithm in another aspect by adding operators to escape from local optimum, but they also didn’t overcome the limitations of the simple GA algorithm. All discussed methods based on the GA algorithm require some parameters determined by the user as motif length. It can be said that the GA algorithm can be enhanced by using a new method that can identify OOPS, ZOOPS, and TCM models, escape from local optimum, improve the fitness function, have good starting positions instead of random initialization, detect multiple motifs with variable lengths, and have intelligent operators in addition to selection, crossover and mutation operators.

PSO is an exploration-exploitation trade off. Exploration is the ability to get the global optimum by search in various regions while the exploitation is the ability to locate the optimum by concentrating the search around a promising candidate solution 159. PSO is a simple concept, easily programmable 160, but it can easily fall in a local optimum and low convergence rate 161.

Based on PSO algorithm, Chang *et al* 144 obtained global optimum in protein sequences when the results were compared with the PROSITE database while Hardin *et al*’s algorithm 112 suffers from local solution. The methods in Lei *et al* 107,108 were tested on both simulated (PMP) and real biological data (*E. coli*); they are efficient and accurate in motif discovery, but they suffer from a long time delay due to full scan on all sequences to check the value of gbest and the repeat -based method was used to automatically terminate the program. PMbPSO algorithm is tested on simulated and real biological datasets 162; the results indicate that PMbPSO algorithm performs better than MbGA and PbGA 163 and it is able to find longer size motifs with a minimum number of mismatches. LPBS algorithm searches for motifs based on reference set. LPBS was tested on Genbank (COI) and there were only two selected species; BosTaurus (Cow-10 DNA sequences with lengths between 658 and 715 *bp*) and GallusGallus (Chicken-9 DNA sequences with lengths between 537 and 699 *bp*). The longest motifs which are able to discover LPBS algorithm are 294 and 261 *bp* in BosTaurus and GallusGallus, respectively. The authors did not evaluate the LPBS method with other previous algorithms of motif discovery. GSA-PSO algorithm was tested on synthetic 39 and real data (TRANSFAC) 164 and the authors reported that the GSA-PSO algorithm performs better than AlignACE, MEME, and GALF 165. GSA-PSO algorithm was compared with old algorithms, although there are a lot of recent algorithms for motif discovery. The presented methods based on the PSO algorithm have some limitations and the methods proposed by Reddy *et al* and Elewa *et al* 4,113 used very simple fitness function and the program terminated manually by determining the numbers of iterations by the user. All the PSO-based methods start with random initialization except the method proposed by Abdullah *et al* 105; this leads to time consuming operations and may not provide any correct solution. Moreover, PSO-based algorithms detect the motifs with OOPS model and ignore ZOOPS, and TCM models, and require motif length as the user input.

Few methods based on ABC, ACO, and CS are the most recent techniques in motif discovery.

ABC algorithm is a popular stochastic algorithm due to it is simple mechanism, and it requires few parameters which is easy to implement 166. Based on ABC algorithm, MO-ABC/DE algorithm is the same as the multiobjective ABC algorithm except for the generation of new candidate solutions; the DE operator was used to generate new candidates that combine existing ones according to a set of simple crossover-mutation schemes. The consensus ABC algorithm, an-other example of ABC technique, was tested on three different data sets and compared with a GA-based tech-nique (MOGAMOD), a DE-based technique (DEPT) 167, and a genetic operator-based ABC algorithm (MO-ABC) 168. Consensus ABC algorithm achieves the highest similarity values of the motifs with different lengths; it used the neighbor selection strategy based on similarity values between consensus sequences and this improved the probability of the selection of a food source.

ACO algorithm has some advantages as; it usually avoids fault convergence due to distributed computation and can be used in dynamic applications.

From ACO algorithm, it can be concluded that there are some disadvantages: (1) It can easily trap local optimum, (2) The consuming time is uncertain, *i.e*. it may take short/long time to converge, (3) The code is not obvious, (4) The ants must visit all points to get a good result that is unsuitable for motif discovery as it takes long time to check every substring in the input sequences and finally, (5) ACO requires many parameters. Based on ACO, Machhi *et al* 117 reported that the total required computing time is reduced.

Finally, there are several advantages for CS algorithm: (1) It usually converges to the global optimality, (2) It combines local and global capabilities and local search takes a quarter of the total search time and the remaining time is for global search which makes CS algorithm more efficient on the global scale, (3) Levy flight is used in its global search instead of standard random walks, so the CS can explore the search space more efficiently, and (4) It is easy to implement, compared with another metaheuristic search which essentially depends on only a single parameter (pa). In a previous study, it was reported that the CSO and MACS can be effectively used to obtain global optimum motif patterns for all used input sequences and they are faster than other nature inspired algorithms 120. Recently, Grey Wolf Optimization for motif finding (GWOMF) 169 was applied to get motifs in DNA input sequences.

## Conclusion

The motif discovery algorithms are classified into four classes of enumerative, probability, nature inspired and combinatorial ones and each one has many subclasses. The comparison between them is listed in table 3. The enumerative technique is an exhaustive search with a simple concept, and it is the only technique that ensures to find all motifs (Except weak motifs). However, it is very slow, and requires a lot of parameters; as a result, it becomes difficult to deal with either long motifs or big data. Moreover, the degenerative positions are limited because of restricted representation of motifs.

**Table 3.** Comparison of approaches

	**Enum.**	**Prob.**	**Nat.**	**Com.**

**Solution technique**	**Exhaustive**	**Heuristic**	**Meta-heuristic**	**?**
**Can deal with a big data set**	×	✓	✓	?
**Is it fast?**	×	✓	?	?
**Can it find long motif?**	×	✓	✓	✓
**Is it a global search?**	✓	×	✓	?
**Low number of required parameters**	×	✓	?	?
**Representation**	Consensus	PWM	Flexible	Flexible
**Can it find all motifs?**	✓	×	?	?
**Can it find weak motifs?**	×	✓	?	?
**Degenerate positions**	Limited	Flexible	Flexible	?
**Objective function**	Flexible	Flexible	Flexible	Flexible
**Is it a simple concept?**	✓	×	✓	?
**Derivation-free mechanism**	×	×	✓	?

Hint: Question mark means maybe yes or no, Enum., prob., Nat., and Com. are standing for Enumerative, Probabilistic, Nature-inspired and Combinatorial, respectively.

Probability approach overcomes many weak points of the enumerative approach like speed, dealing with long motifs and big data, numbers of required parameters and degenerated positions, and can find weak motifs. But the probability approach is a complex concept and can’t find all motifs.

The third category, natured inspired approach, combines the main features of the first two approaches.

This approach is a simple concept and it is a global search but at the same time can deal with the big data and long motifs. It has a flexible representation of motifs and this lead to an unlimited number of degenerated positions.

The last category is the combinatorial approach; its ability depends on the hybrid algorithms that combine to form the required algorithm.

The common features of all algorithms are the flexibility of objective function.

The presented classification of motif discovery algorithms is useful to get a general overview and to build a good motif discovery algorithm.

From various suggested methods for motif discovery problem, a good tool for motif discovery can be built. The tool must contain these features: (1) It should identify all models, *i.e*. OOPS, ZOOPS, TCM, (2) It should possess global search ability, (3) It should optimize the scoring function, (4) Parallel processing ability is a necessity, (5) It should have optimized data structures, (6) It should be able to detect long and short motifs, (7) It should have the capacity of multiple motif discoveries at the same time, *i.e*. without removing the discovered motif to find the next, (8) And multiple motifs discovery with variable lengths, and (9) It needs to have an automatic system by decreasing the number of required parameters determined by the user. The next phase of this work is to develop a new motif discovery algorithm that combines the main features of enumerative and probabilistic approaches and to use it as a seed to a nature-inspired algorithm by considering the factors mentioned above.
